# Pre-treatment neutrophil to lymphocyte ratio predicts the chemoradiotherapy outcome and survival in patients with oral squamous cell carcinoma: a retrospective study

**DOI:** 10.1186/s12885-016-2079-6

**Published:** 2016-01-26

**Authors:** Hikaru Nakashima, Yuichiro Matsuoka, Ryoji Yoshida, Masashi Nagata, Akiyuki Hirosue, Kenta Kawahara, Junki Sakata, Hidetaka Arita, Akimitsu Hiraki, Hideki Nakayama

**Affiliations:** Department of Oral and Maxillofacial Surgery, Graduate School of Life Sciences, Kumamoto University, Honjo 1-1-1, Chuo-ku, Kumamoto 860-8556 Japan; Section of Oral Oncology, Department of Oral and Maxillofacial Surgery, Fukuoka Dental College, 2-15-1 Tamura, Sawara-ku, Fukuoka 814-0193 Japan

**Keywords:** Neutrophil to lymphocyte ratio (NLR), Oral squamous cell carcinoma (OSCC), Chemoradiotherapy, Resistance, Prognosis

## Abstract

**Background:**

The Neutrophil to lymphocyte ratio (NLR) has prognostic value in patients with a variety of cancers. However, its prognostic significance in oral squamous cell carcinoma (OSCC) patients has not been fully explored. The purpose of this study was to determine the clinical significance of NLR in patients with OSCC.

**Methods:**

OSCC patients who underwent surgery following 5-fluorouracil (5-FU)-based chemoradiotherapy were enrolled in this study. The associations between the NLR status and various clinicopathological features were examined, and the effects of the NLR on the prognosis were evaluated. Analysis of circulating interleukin-6 (IL-6) was carried out and correlation with NLR and C-reactive protein concentration (CRP) was examined.

**Results:**

An elevated NLR was significantly correlated with advanced T-stage and poor response to chemoradiotherapy. Moreover, a Cox regression analysis based on the disease-free survival (DFS) revealed the NLR status (hazard ratio, 2.013; *P* = 0.041) and pathological response to chemoradiotherapy (hazard ratio, 0.226; *P* = 0.001) to be significant prognostic factors in OSCC patients. Furthermore, circulating IL-6 was found to correlate with NLR and CRP.

**Conclusion:**

The NLR is a potential biomarker for predicting the clinical response to 5-FU-based chemoradiotherapy and the survival in OSCC patients, and the systemic inflammatory response may be potential target for improving patient’s prognosis.

**Electronic supplementary material:**

The online version of this article (doi:10.1186/s12885-016-2079-6) contains supplementary material, which is available to authorized users.

## Background

Oral cancer is one of the most common cancers [1]. The survival rate of patients with oral cancer has not improved despite improvements and innovations in diagnostic techniques and treatments, and the prognosis of OSCC remains poor, with a 5-year survival rate of approximately 50 % [2]. Therefore, in order to further improve patient outcomes, it is imperative to investigate novel markers for predicting the prognosis and guide the therapeutic management of OSCC.

Increasing evidence suggests that the tumor microenvironment plays an important role in determining the biological behavior of cancers, such as proliferation, invasion, metastasis and therapeutic resistance [3, 4]. In recent years, it has become clear that cancer-associated inflammation, in the form of local and systemic inflammatory responses, is a key factor of disease progression and survival in several cancers [5, 6]. Among various characteristics of the systemic inflammatory response, including the plasma CRP, hypoalbuminemia and Glasgow Prognostic Score (GPS, which combines CRP and albumin), as well as the absolute white cell count and platelet/lymphocyte ratio (PLR), the NLR is one of the most easily measurable parameters of the systemic inflammatory response [7].

It has been demonstrated that an increased pre-treatment NLR is associated with a poor clinical outcome for various types of cancers, including colorectal cancer [7], pancreatic cancer [8], small cell lung cancer [9], breast cancer [10] and diffuse large B-cell lymphoma [11]. Although the clinical significance of the NLR has been revealed in various types of cancer, there are few studies investigating the clinical significance of the NLR in cases of head and neck carcinoma, including OSCC [12, 13]. In particular, the relationship between the NLR and treatment response in patients receiving chemoradiotherapy for OSCC has not been elucidated.

Among various cytokines that mediate the systemic inflammatory response, IL-6 is known to be an important cytokine that stimulates hepatocytes to induce acute-phase proteins, including CRP, and elicits neutrophil proliferation, thereby increasing the NLR [14, 15]. Recently, it has been suggested that targeting for IL-6 mediated signaling is a potential strategy for controlling the systemic inflammatory response [7, 16], as well as the malignant behavior of cancer cells themselves [17]. However, the relationship between the circulating concentration of IL-6 and the status of the systemic inflammatory response in OSCC patients has not yet been fully examined.

Therefore, for the first time, we investigated the clinical significance of the NLR and the correlation between circulating IL-6 and the systemic inflammatory response in OSCC patients treated with preoperative chemoradiotherapy in the present study.

## Methods

### Clinical characteristics of the patients

In total, there were 124 patients with advanced OSCC treated at Kumamoto University Hospital between October 2003 and January 2009 whose clinical information and laboratory parameters were available. Patients with factors that could influence the NLR, such as concurrent infections, chronic inflammatory diseases, recent treatment with steroids or previous treatment with chemo- or radiotherapy were excluded. All patients were treated preoperatively with chemoradiotherapy. Radiotherapy was administered at a daily dose of 2.0 Gy five times a week for 15 days. An oral fluorouracil anti-cancer agent, S-1, was concurrently administered at a dose of 80, 100 or 120 mg/day according to each patient’s body surface area for 14 days from the initiation of radiotherapy. All tumors were staged according to the TNM classification of the UICC (2002), and the degree of differentiation was determined based on the grade classification of the WHO. Using specimens obtained during surgery, the histological response to chemoradiotherapy was graded according to the criteria proposed by Shimosato et al. [18], as follows: grade I, tumor structures are not destroyed; grade IIa, destruction of the tumor structure is mild (i.e., ‘viable tumor cells’ are frequently observed); grade IIb, destruction of the tumor structure is severe (i.e., ‘viable tumor cells’ are few in number); grade III, nonviable tumor cells are present and grade IV, no tumor cells remain. The current study followed the guidelines of the Ethics Committee of Kumamoto University. The nature and aims of the study were explained to all patients, who gave their written informed consent for the research.

### Follow-up

Patients underwent hematologic tests and assessments of clinical symptoms every 2 weeks. The presence of a recurrence was determined by means of imaging modalities, including CT, MRI, US, and PET-CT. The patients underwent at least one type of imaging examination at intervals of 3–4 months during the first 2 years after surgery, and at intervals of 4–6 months thereafter until 5 years after surgery.

### Methods

Blood samples were collected before the administration of preoperative chemoradiotherapy for a routine laboratory analysis of the full blood count, neutrophil count and lymphocyte count. Briefly, the NLR was determined by dividing the absolute neutrophil count by the absolute lymphocyte count, and the NLR data were then dichotomized and divided into two groups as NLR-low and -high. The value of NLR that best discriminated between good and poor survival, with the most significant *P*-value according to the log-rank test, was determined by testing all possible cutoffs. Peripheral blood samples available for the assessment of the circulating concentrations of IL-6 were collected from only 37 patients in our present cohort. The blood samples for circulating IL-6 measurement were centrifuged and the serum was stored at -80 °C before the analysis of IL-6. The circulating concentrations of IL-6 were measured using a Human IL-6 Quantikine ELISA Kit (R&D Systems, MN, US) according to the manufacturer’s instructions. This study was approved by the ethics committee of Kumamoto University.

### Statistical analysis

The chi-square test was used to determine the associations between the pre-treatment NLR status and the clinical and pathological variables. Overall survival (OS) and DFS were defined as the time from treatment initiation (chemoradiotherapy) to the date of death from any cause and the date of recurrence of cancer or death from any cause, respectively. The Kaplan–Meier method was used to estimate the probability of OS and DFS as a function of time, and the statistical differences in the survival of the subgroups of patients were compared using the log-rank test. A multivariate survival analysis was performed using the Cox regression model to study the effects of the pre-treatment NLR status on the DFS. Scatter plots were used to observe the associations between the circulating concentrations of IL-6 and NLR or CRP, and relationships between these parameters were investigated with Pearson’s correlation coefficient test. All *P-*values were based on two-tailed statistical analyses, and *P*-values of < 0.05 were considered to be statistically significant (*P < 0.05 and ** *P* < 0.01). The statistical analyses were completed using the JMP 9 software program (SAS Institute Inc., Cary, NC).

## Results

### Relationships between the pre-treatment NLR status and clinicopathological characteristics

In order to elucidate the clinical significance of the pre-treatment NLR in OSCC patients treated with preoperative 5-FU-based chemoradiotherapy, we examined the correlations between the NLR status and the clinicopathological variables. The median neutrophil count was 4200 per mm^3^, the median lymphocyte count was 1770 per mm^3^ and the median NLR was 2.4 (95 % CI = 2.0–2.8). When the NLR was analyzed as a dichotomous variable, a cut-off point of 2.4 provided the strongest prognostic value in our dataset, which included the average value (=2.7) and the tertiles (Additional file 1: Figure S1 and Additional file 2: Figure S2). This level was therefore chosen for further study. The chosen cut-off point was considerably close to the OSCC data that were previously reported by Fang et al. [12]. The patients were further divided into two groups depending on the NLR status: 64 patients (51.6 %) had an NLR of ≥ 2.4 at baseline (NLR-high), whereas 60 had a lower NLR (NLR-low). Table 1 shows the distribution of the clinical background characteristics of the studied patients divided into the two groups (NLR-high and NLR-low) according to the NLR. The frequency of NLR-high patients was significantly higher among the cases who showed an advanced T-stage and poor pathological response to preoperative chemoradiotherapy (*P* = 0.016 and *P* = 0.038). There were no differences in the expression status of NLR according to age, gender, primary tumor site, N-stage, clinical stage or differentiation. These results indicate that the NLR status is associated with the biological behavior of cancer cells, especially tumor growth and resistance to preoperative chemoradiotherapy.

**Table 1:** Correlation between the NLR status and clinicopathological factors in 124 OSCC patients

Characteristics	Total	NLR status		*P*-value
		High	Low	
		n (%)	n (%)	
	124	64 (51.6)	60 (48.4)	
Age (years)				
Median	67.2	68.8	65.4	
Range	28–87	39–85	28–87	
≤ 65	51	22 (43.1)	29 (58.9)	0.114
> 65	73	42 (57.5)	31 (42.5)	
Gender				
Male	75	38 (50.7)	37 (49.3)	0.794
Female	49	26 (53.1)	23 (46.9)	
Primary site				
Tongue	35	18 (51.4)	17 (48.6)	0.958
Mandible	42	24 (57.1)	18 (42.9)	
Maxilla	18	8 (44.4)	10 (55.6)	
Oral floor	15	7 (46.7)	8 (53.3)	
Buccal mucosa	14	7 (50.0)	7 (50.0)	
T-stage				
T1, T2	39	13 (33.3)	26 (66.6)	0.016*
T3	38	21 (55.3)	17 (44.7)	
T4	47	30 (63.8)	17 (36.2)	
N-stage				
N0, N1	25	13 (52.0)	12 (48.0)	0.965
≥ N2	99	51 (51.5)	48 (48.5)	
Clinical stage				
III	38	20 (52.6)	18 (47.4)	0.880
IV	86	44 (51.2)	42 (48.8)	
Differentiation				
Well	99	49 (49.5)	50 (50.5)	0.348
Moderate	25	15 (60.0)	10 (40.0)	
Pathological response				
Grade ≤ IIb (poor ~ partial response)	51	32 (62.7)	19 (37.3)	0.038*
Grade ≥ III (complete response)	73	32 (43.8)	41 (56.2)	

*Abbreviation: NLR* neutrophil to lymphocyte ratio, *OSCC* oral squamous cell carcinoma. The chi-square test was used to examine the relationships between NLR status and clinicopathologic factors

*, *P* < 0.05

#### Relationships between the pre-treatment NLR status and survival time

**Fig. 1:**
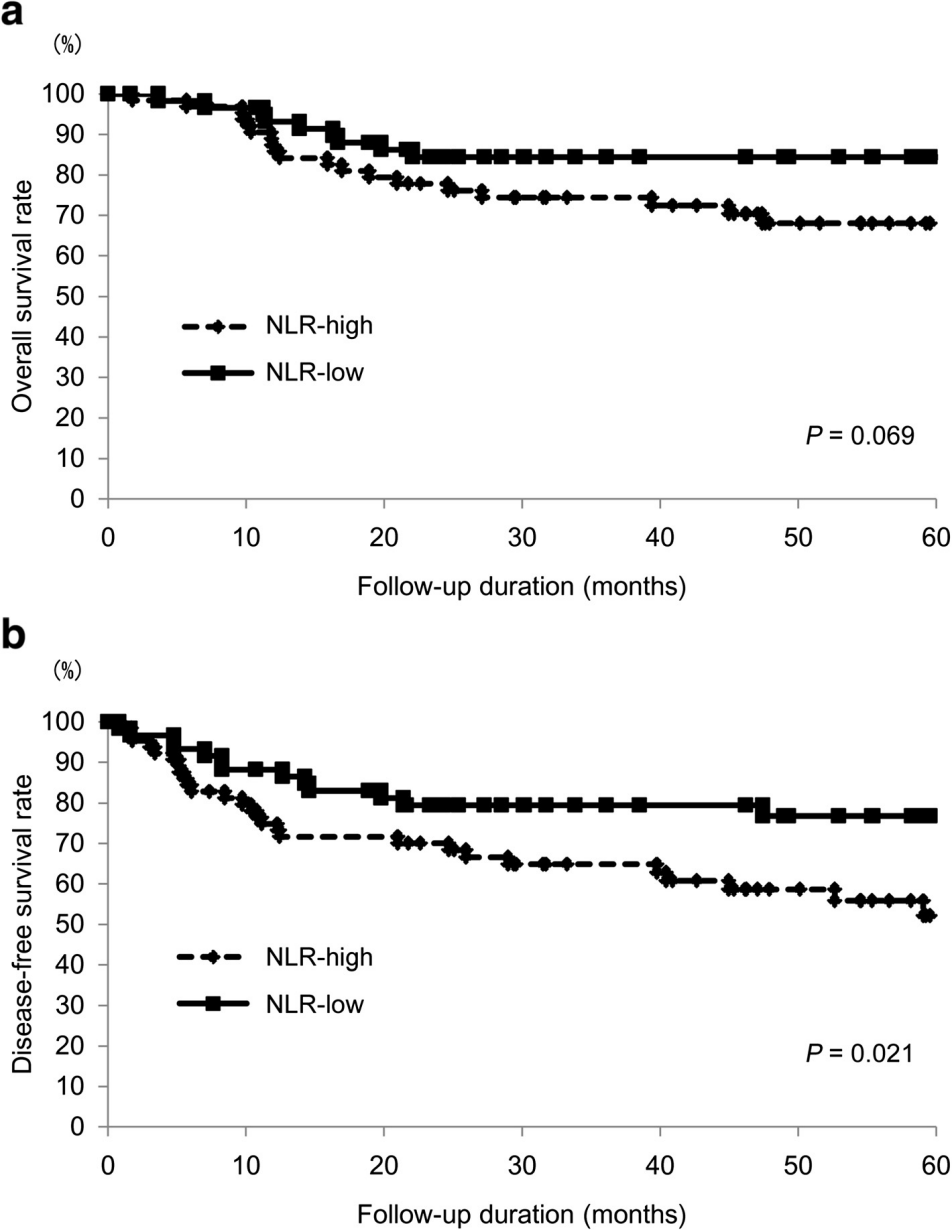
Relationships between the NLR and cancer-specific survival in patients with OSCC. In the Kaplan-Meier survival analysis of patients with oral squamous cell carcinoma (OSCC), the patients were divided into two groups based on the NLR status (low and high groups). **a** Overall survival (OS) of the 124 OSCC patients based on the NLR status. *, *P* < 0.05. **b** Disease-free survival (DFS) of the 124 OSCC patients based on the NLR status. *, *P* < 0.05

In order to assess the relationships between the pre-treatment NLR status and survival time, the overall and disease-free survival of the 124 OSCC patients were analyzed using the Kaplan-Meier method. The median OS from the time of diagnosis in the follow-up period was 47.5 months (95 % CI = 39.2–55.9), while the median DFS in the follow-up period was 40.1 months (95 % CI = 33.0–47.2). Twenty-eight patients died during the follow-up period. The median OS in the NLR-high group during the follow-up period was 46.2 months (95 % CI = 34.9–57.5), while that in the NLR-low group was 51.1 months (95 % CI = 38.2–64.1) (*P* = 0.069; Fig. 1a). The 5-year OS rate of the NLR-high group tended to be lower than that of the NLR-low group; however, there were no significant differences between the two groups. The median DFS in the NLR-high group during the follow-up period was 36.5 months (95 % CI = 27.6–45.5), while that in the NLR-group was 46.8 months (95 % CI = 35.0–58.7) (*P* = 0.021; Fig. 1b). The 5-year DFS rate in the NLR-high group was significantly lower than that in the NLR-low group. Collectively, our data indicate that the status of NLR may be a potential prognostic factor in patients with OSCC receiving 5-FU-based chemoradiotherapy.

### Multivariate analysis of prognostic factors

In order to determine the independent prognostic value of the pre-treatment NLR for DFS, a multivariate analysis was performed using a Cox proportional hazards regression model. After adjusting for the primary site, T-stage, N-stage, tumor cell differentiation and pathological response to chemoradiotherapy, the influence of the NLR on DFS (hazard ratio = 2.013, 95 % CI = 1.029–4.144; *P* = 0.041) remained. In addition, the pathological response to preoperative chemoradiotherapy was also a significant prognostic factor (hazard ratio, 0.226; 95 % CI, 0.093–0.527; *P* = 0.001) (Table 2). Therefore, we assumed that the NLR status, as well as the pathological response to preoperative chemoradiotherapy, potentially affects the prognosis and is a potential independent prognostic factor in OSCC patients receiving 5-FU-based chemoradiotherapy.

**Table 2:** The results of a multivariate regression analysis for predicting the disease-free survival of 124 OSCC patients

Variables	Assigned score	Hazard ratio (95 % CI)	*P*-value
Primary site			
Tongue	1	2.240 (0.924–5.267)	0.073
Mandible	2		
Maxilla	3		
Oral floor	4		
Buccal mucosa	5		
T-stage			
T1, T2	1	0.942 (0.419–2.132)	0.885
T3	2		
T4	3		
N-stage			
N0, N1	0	1.844 (0.701–5.063)	0.217
≥ N2	1		
Differentiation			
Well	0	0.636 (0.267–1.348)	0.249
Moderate	1		
Pathological response			
Grades 0, I, IIa	1	0.226 (0.093–0.527)	0.001*
Grade IIb	2		
Grade III	3		
Grade IV	4		
NLR status			
Low	0	2.013 (1.029–4.144)	0.041*
High	1		

*Abbreviations: CI* confidence interval, *NLR* neutrophil to lymphocyte ratio, *OSCC* oral squamous cell carcinoma

*, *P* < 0.05

### Relationships between the circulating concentration of IL-6 and pre-treatment markers of the systemic inflammatory response

**Fig. 2:**
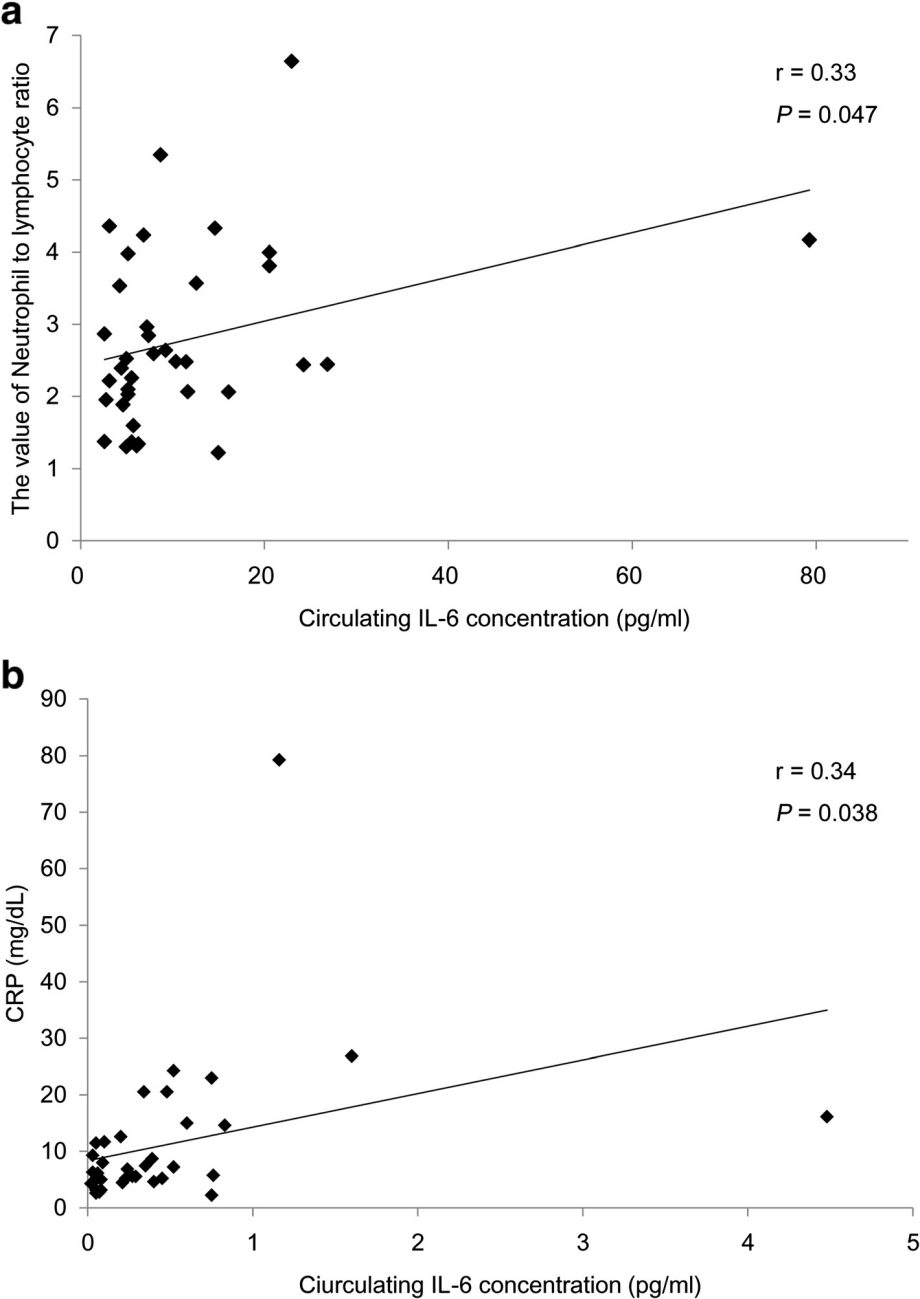
Relationship between the circulating concentrations of IL-6 and the pre-treatment NLR and CRP in the 37 patients with OSCC. Scatter plots of the circulating IL-6 concentration and indicators of the systemic inflammatory response. **a** Relationship between the circulating concentrations of IL-6 and the pre-treatment NLR. The x-axis indicates the circulating IL-6 concentration and the y-axis shows the value of NLR. The correlation was investigated using Pearson’s correlation coefficient test. *, *P* < 0.05. **b** Relationship between the circulating concentrations of IL-6 and the pre-treatment CRP. The x-axis indicates the circulating IL-6 concentration and the y-axis shows the value of CRP. The correlation was investigated using Pearson’s correlation coefficient test. *, *P* < 0.05

In order to explore the potential relationships between the circulating IL-6 levels and systemic inflammatory response-related characteristics, such as NLR and CRP, in the OSCC patients, the circulating concentrations of IL-6 in pre-treatment serum were measured using ELISA and examined the correlations with the NLR and CRP values. On Pearson’s correlation coefficient test, there were significant associations between the circulating concentrations of IL-6 and the NLR (Fig. 2a, *r* = 0.33, *P* = 0.047) and CRP (Fig. 2b, *r* = 0.34, *P* = 0.038). These results suggest that the circulating IL-6 level may have the potential to mediate the systemic inflammatory response in OSCC patients.

## Discussion

In the present study, we found three major findings. First, an increased pre-treatment NLR was correlated with the T-stage and resistance to 5-FU-based chemoradiotherapy in patients with OSCC. Second, an increased pre-treatment NLR was correlated with a shorter DFS in the OSCC patients who underwent preoperative 5-FU-based chemoradiotherapy. Finally, there was a positive correlation between the circulating IL-6 concentration in the peripheral blood and the NLR value. To the best of our knowledge, this is the first study to describe the use of the NLR in the OSCC setting in patients receiving 5-FU-based chemoradiotherapy to provide useful information regarding resistance to chemoradiotherapy and prognostication. Furthermore, a positive correlation between the circulating IL-6 and the systemic inflammatory response in OSCC was demonstrated for the first time.

There are several explanations for the relationship between an elevated NLR and aggressive phenotypes in cases of cancer. Generally, an elevated NLR reflects an increased neutrophil count and/or decreased lymphocyte count. Circulating neutrophils contain and secrete various cytokines, chemokines, proteases and growth factors, including vascular endothelial growth factor [19], platelet-derived growth factor, fibroblast growth factor [20], matrix metalloproteinase [21] and IL-6 [22]. These molecules create a microenvironment for extracellular matrix remodeling, endothelial cell migration and tumor cell dissociation [23]. Moreover, it has been shown that neutrophils may suppress the cytolytic activity of a variety of immune cells, such as lymphocytes, activated T cells and natural killer cells under the co-culture conditions of neutrophils and lymphocytes obtained from healthy donors, and the degree of suppression is closely associated with the number of neutrophils [24, 25]. Collectively, an elevated NLR may be associated with establishment of the tumor microenvironment and low immunocompetence in cancer patients with OSCC as well as subsequently induced tumor growth [4]. These observations and hypothesis strongly support our results showing that an elevated NLR (i.e., elevated systemic inflammatory response) contributes to tumor progression in patients with OSCC.

Recently, the clinical significance of the NLR in patients receiving chemotherapy as well as surgical resection has been reported. Chua et al. [7] reported the use of the NLR as a marker of the systemic inflammatory response and an independent predictor of the clinical benefit, progression and survival in patients receiving chemotherapy for metastatic colorectal cancer. Santoni et al. [26] revealed that the NLR is an independent prognostic factor for metastatic renal cell carcinoma among patients treated with molecular targeted therapy. In cases of advanced non-small cell lung cancer and high-grade serous epithelial ovarian cancer, the relationship between an elevated NLR and a poor response to chemotherapy was also recently reported [27, 28]. The results of the present study are in line with those of these previous data and suggest that the pre-treatment NLR is potentially a useful predictor of the response to 5-FU-based chemoradiotherapy. In addition, our data indicate the presence of chemoradioresistance mechanisms mediated by the systemic inflammatory response in OSCC as well as other malignancies.

Notably, an elevated NLR in OSCC patients is significantly correlated not only with a poor response to chemoradiotherapy, but also a shorter DFS (Fig. 1b). Although the reason why the NLR was not found to correlate with the OS is unclear, the potential correlation between the NLR and non-malignant diseases, such as myocardial infarction, in which the systemic inflammation response has been implicated as a major contributing factor [29], may possibly affect our results. On the other hand, because the NLR is significantly associated with the pathological response to preoperative chemoradiotherapy, the difference in the survival rate in DFS may be partly explained by differences in the treatment response to preoperative chemoradiotherapy between the low and high NLR groups. Consistent with our results, it has been reported that an elevated NLR is correlated with a worse prognosis in many malignancies [7–11]. Moreover, Fang et al. [12] reported that an elevated NLR is significantly associated with a worse disease-free and overall survival in OSCC patients. Nevertheless, to the best of our knowledge, there are no previous studies investigating patients receiving chemoradiotherapy. This is the first report of the association between the NLR status and the prognosis in patients with OSCC receiving chemoradiotherapy. In the setting of OSCC, treatment options combining anticancer agents or chemoradiotherapy, including preoperative therapies, are important treatment strategies [30–32], and investigating good prognostic markers in patients receiving these treatment modalities is thus valuable. The results of the present study suggest that the NLR may be useful for making treatment decisions in OSCC patients receiving chemo- and/or chemoradiotherapy. Because the NLR is a simple, readily available and robust laboratory variable, we assume that this concept may be widely accepted in various types of cancer and in various countries.

Although the molecular mechanisms of therapeutic resistance have not been fully elucidated, the pre-existing state of the tumor microenvironment established by the systemic inflammatory response may provide cancer cells with advanced malignant phenotypes and immune suppressive conditions, which could determine the response to chemotherapy. Recently, it has been suggested that IL-6, which is derived from cancer cells as well as from tumor stromal cells, such as cancer-associated fibroblasts and infiltrating immune cells [7, 33], may modulate both the local tumor microenvironment and the chronic systemic inflammatory response by causing the differentiation of tumor-infiltrating monocytes into immunosuppressive, M2-like macrophages [34] and by attracting regulatory T cells [35] in patients with cancer, thereby allowing cancer cells to acquire an advanced malignant phenotype. In OSCC, we previously demonstrated that tocilizumab, a humanized anti-IL-6R antibody, may be effective for OSCC treatment, at a minimum by inhibiting angiogenesis and lymphangiogenesis *in vitro* and *vivo* [17, 36]. Taken together, our findings suggest that IL-6 is a potential treatment target for OSCC, improving the outcomes of chemoradiotherapy and prognosis of patients with OSCC by operating on the local and systemic inflammatory response initiated by tumor- and/or stromal cell-derived IL-6. Therefore, the suppression of circulating IL-6 levels with tocilizumab may enhance the immune response and sensitize OSCC cells to chemoradiotherapy, thereby resulting in an improvement of the clinical outcomes of patients with refractory OSCC.

There are some limitations associated with this study. First, this was a retrospective study, which is susceptible to bias in both data selection and analysis. Second, the NLR differs among individuals and may be influenced by general conditions and drugs that could not be accounted for in this study. Third, the present data were obtained from patients who were treated with modest doses of 5-FU-based chemoradiotherapy and surgery. Further investigation will therefore be needed to determine whether our results can be applied to all OSCC patients. Despite these limitations, our findings suggest that an elevated NLR contributes to resistance to chemo- and/or chemoradiotherapy and a poor prognosis in patients with OSCC. In addition, the status of the NLR may be useful for making treatment decisions to improve the survival of OSCC patients. Further studies are needed to determine the resistance mechanisms of chemoradiotherapy underlying an elevated NLR and to adequately assess the potential role of NLR in guiding treatment decisions. Furthermore, the therapeutic efficacy of targeting IL-6 must be assessed to overcome chemoradioresistance and confirm the clinical significance of our findings.

## Conclusions

Our findings reported herein demonstrated that pre-treatment NLR is a potential biomarker for predicting theclinical response to 5-FU-based chemoradiotherapy and the survival in OSCC patients, and the systemicinflammatory response may be potential target for improving patient's prognosis.

## Additional files

Additional file 1: Figure S1. The relationships between the NLR status and cancer-specific survival in patients with OSCC. In the Kaplan-Meier survival analysis of patients with oral squamous cell carcinoma (OSCC), the patients were divided into two groups (low and high groups) based on the average NLR value (=2.7). (A) Overall survival (OS) of the 124 OSCC patients based on their NLR status. (B) Disease-free survival (DFS) of the 124 OSCC patients based on their NLR status. (JPG 867 kb) Additional file 2: Figure S2. The relationship between the NLR status and cancer-specific survival in patients with OSCC. In the Kaplan-Meier survival analysis of patients with oral squamous cell carcinoma (OSCC), the patients were divided into three groups based on their NLR status (Tertiles 1, 2 and 3). (A) The overall survival (OS) of the 124 OSCC patients stratified by their NLR status. (B) The disease-free survival (DFS) of the 124 OSCC patients stratified by their NLR status. (JPG 974 kb)

## Abbreviations

NLR: neutrophil to lymphocyte ratio
OSCC: oral squamous cell carcinoma
5-FU: 5-fluorouracil
IL-6: interleukin-6
CRP: C-reactive protein concentration
DFS: disease-free survival
GPS: Glasgow Prognostic Score
PLR: platelet/lymphocyte ratio
OS: overall survival

## References

[1] Siegel R, Naishadham D, Jemal A (2012). Cancer statistics, 2012. CA Cancer J Clin.

[2] Gupta S, Kong W, Peng Y, Miao Q, Mackillop WJ (2009). Temporal trends in the incidence and survival of cancers of the upper aerodigestive tract in Ontario and the United States. Int J Cancer.

[3] Meads MB, Gatenby RA, Dalton WS (2009). Environment-mediated drug resistance: a major contributor to minimal residual disease. Nat Rev Cancer.

[4] Hanahan D, Weinberg RA (2011). Hallmarks of cancer: the next generation. Cell.

[5] McMillan DC (2009). Systemic inflammation, nutritional status and survival in patients with cancer. Curr Opin Clin Nutr Metab Care.

[6] Diakos CI, Charles KA, McMillan DC, Clarke SJ (2014). Cancer-related inflammation and treatment effectiveness. Lancet Oncol.

[7] Chua W, Charles KA, Baracos VE, Clarke SJ (2011). Neutrophil/lymphocyte ratio predicts chemotherapy outcomes in patients with advanced colorectal cancer. Br J Cancer.

[8] An X, Ding PR, Li YH, Wang FH, Shi YX, Wang ZQ (2010). Elevated neutrophil to lymphocyte ratio predicts survival in advanced pancreatic cancer. Biomarkers.

[9] Kang MH, Go SI, Song HN, Lee A, Kim SH, Kang JH (2014). The prognostic impact of the neutrophil-to-lymphocyte ratio in patients with small-cell lung cancer. Br J Cancer.

[10] Azab B, Bhatt VR, Phookan J, Murukutla S, Kohn N, Terjanian T (2012). Usefulness of the neutrophil-to-lymphocyte ratio in predicting short- and long-term mortality in breast cancer patients. Ann Surg Oncol.

[11] Porrata LF, Ristow K, Habermann T, Inwards DJ, Micallef IN, Markovic SN (2010). Predicting survival for diffuse large B-cell lymphoma patients using baseline neutrophil/lymphocyte ratio. Am J Hematol.

[12] Fang HY, Huang XY, Chien HT, Chang JT, Liao CT, Huang JJ (2013). Refining the role of preoperative C-reactive protein by neutrophil/lymphocyte ratio in oral cavity squamous cell carcinoma. Laryngoscope.

[13] Rassouli A, Saliba J, Castano R, Hier M, Zeitouni AG (2015). Systemic inflammatory markers as independent prognosticators of head and neck squamous cell carcinoma. Head Neck.

[14] Ohsugi Y (2007). Recent advances in immunopathophysiology of interleukin-6: an innovative therapeutic drug, tocilizumab (recombinant humanized anti-human interleukin-6 receptor antibody), unveils the mysterious etiology of immune-mediated inflammatory diseases. Biol Pharm Bull.

[15] Ruscetti FW (1994). Hematologic effects of interleukin-1 and interleukin-6. Curr Opin Hematol.

[16] Scheller J, Ohnesorge N, Rose-John S (2006). Interleukin-6 trans-signalling in chronic inflammation and cancer. Scand J Immunol.

[17] Shinriki S, Jono H, Ota K, Ueda M, Kudo M, Ota T (2009). Humanized anti-interleukin-6 receptor antibody suppresses tumor angiogenesis and in vivo growth of human oral squamous cell carcinoma. Clin Cancer Res.

[18] Shimosato Y, Oboshi S, Baba K (1971). Histological evaluation of effects of radiotherapy and chemotherapy for carcinomas. Jpn J Clin Oncol.

[19] Di Carlo E, Forni G, Musiani P (2003). Neutrophils in the antitumoral immune response. Chem Immunol Allergy.

[20] Colotta F, Allavena P, Sica A, Garlanda C, Mantovani A (2009). Cancer-related inflammation, the seventh hallmark of cancer: links to genetic instability. Carcinogenesis.

[21] Deryugina EI, Zajac E, Juncker-Jensen A, Kupriyanova TA, Welter L, Quigley JP (2014). Tissue-infiltrating neutrophils constitute the major in vivo source of angiogenesis-inducing MMP-9 in the tumor microenvironment. Neoplasia.

[22] Ericson SG, Zhao Y, Gao H, Miller KL, Gibson LF, Lynch JP (1998). Interleukin-6 production by human neutrophils after Fc-receptor cross-linking or exposure to granulocyte colony-stimulating factor. Blood.

[23] Dumitru CA, Lang S, Brandau S (2013). Modulation of neutrophil granulocytes in the tumor microenvironment: mechanisms and consequences for tumor progression. Semin Cancer Biol.

[24] El-Hag A, Clark RA (1987). Immunosuppression by activated human neutrophils. Dependence on the myeloperoxidase system. J Immunol.

[25] Shau HY, Kim A (1988). Suppression of lymphokine-activated killer induction by neutrophils. J Immunol.

[26] Santoni M, De Giorgi U, Iacovelli R, Conti A, Burattini L, Rossi L (2013). Pre-treatment neutrophil-to-lymphocyte ratio may be associated with the outcome in patients treated with everolimus for metastatic renal cell carcinoma. Br J Cancer.

[27] Botta C, Barbieri V, Ciliberto D, Rossi A, Rocco D, Addeo R (2013). Systemic inflammatory status at baseline predicts bevacizumab benefit in advanced non-small cell lung cancer patients. Cancer Biol Ther.

[28] Koti M, Siu A, Clement I, Bidarimath M, Turashvili G, Edwards A (2015). A distinct pre-existing inflammatory tumour microenvironment is associated with chemotherapy resistance in high-grade serous epithelial ovarian cancer. Br J Cancer.

[29] Nunez J, Nunez E, Bodi V, Sanchis J, Minana G, Mainar L (2008). Usefulness of the neutrophil to lymphocyte ratio in predicting long-term mortality in ST segment elevation myocardial infarction. Am J Cardiol.

[30] Vermorken JB, Remenar E, van Herpen C, Gorlia T, Mesia R, Degardin M (2007). Cisplatin, fluorouracil, and docetaxel in unresectable head and neck cancer. N Engl J Med.

[31] Kirita T, Yamanaka Y, Imai Y, Yamakawa N, Aoki K, Nakagawa Y (2012). Preoperative concurrent chemoradiotherapy for stages II-IV oral squamous cell carcinoma: a retrospective analysis and the future possibility of this treatment strategy. Int J Oral Maxillofac Surg.

[32] Freier K, Engel M, Lindel K, Flechtenmacher C, Muhling J, Hassfeld S (2008). Neoadjuvant concurrent radiochemotherapy followed by surgery in advanced oral squamous cell carcinoma (OSCC): a retrospective analysis of 207 patients. Oral Oncol.

[33] Sun X, Mao Y, Wang J, Zu L, Hao M, Cheng G, et al. IL-6 secreted by cancer-associated fibroblasts induces tamoxifen resistance in luminal breast cancer. Oncogene. 2014. 10.1038/onc.2014.158 24909173

[34] Dijkgraaf EM, Heusinkveld M, Tummers B, Vogelpoel LT, Goedemans R, Jha V (2013). Chemotherapy alters monocyte differentiation to favor generation of cancer-supporting M2 macrophages in the tumor microenvironment. Cancer Res.

[35] Preston CC, Maurer MJ, Oberg AL, Visscher DW, Kalli KR, Hartmann LC (2013). The ratios of CD8+ T cells to CD4 + CD25+ FOXP3+ and FOXP3- T cells correlate with poor clinical outcome in human serous ovarian cancer. PLoS One.

[36] Shinriki S, Jono H, Ueda M, Ota K, Ota T, Sueyoshi T (2011). Interleukin-6 signalling regulates vascular endothelial growth factor-C synthesis and lymphangiogenesis in human oral squamous cell carcinoma. J Pathol.

