# Supplementary figures and images for: Pre-treatment neutrophil to lymphocyte ratio predicts the chemoradiotherapy outcome and survival in patients with oral squamous cell carcinoma: a retrospective study
Source: BMC Cancer. 2016 Jan 26;16:41. doi: 10.1186/s12885-016-2079-6 (PMC4728793; doi:10.1186/s12885-016-2079-6)

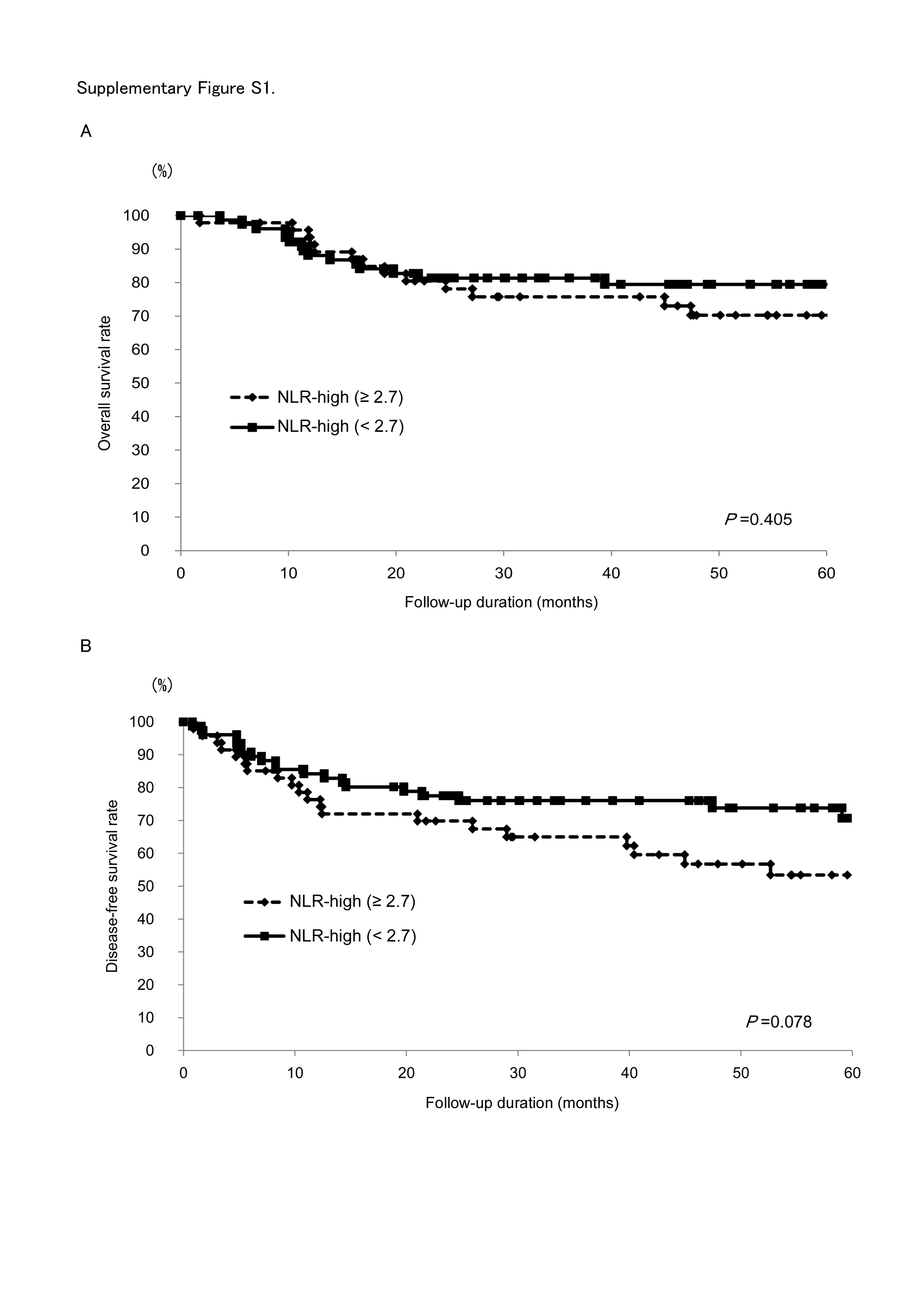

Supplement: Additional file 1: Figure S1. — The relationships between the NLR status and cancer-specific survival in patients with OSCC. In the Kaplan-Meier survival analysis of patients with oral squamous cell carcinoma (OSCC), the patients were divided into two groups (low and high groups) based on the average NLR value (=2.7). (A) Overall survival (OS) of the 124 OSCC patients based on their NLR status. (B) Disease-free survival (DFS) of the 124 OSCC patients based on their NLR status. (JPG 867 kb) [file 12885_2016_2079_MOESM1_ESM.jpg]

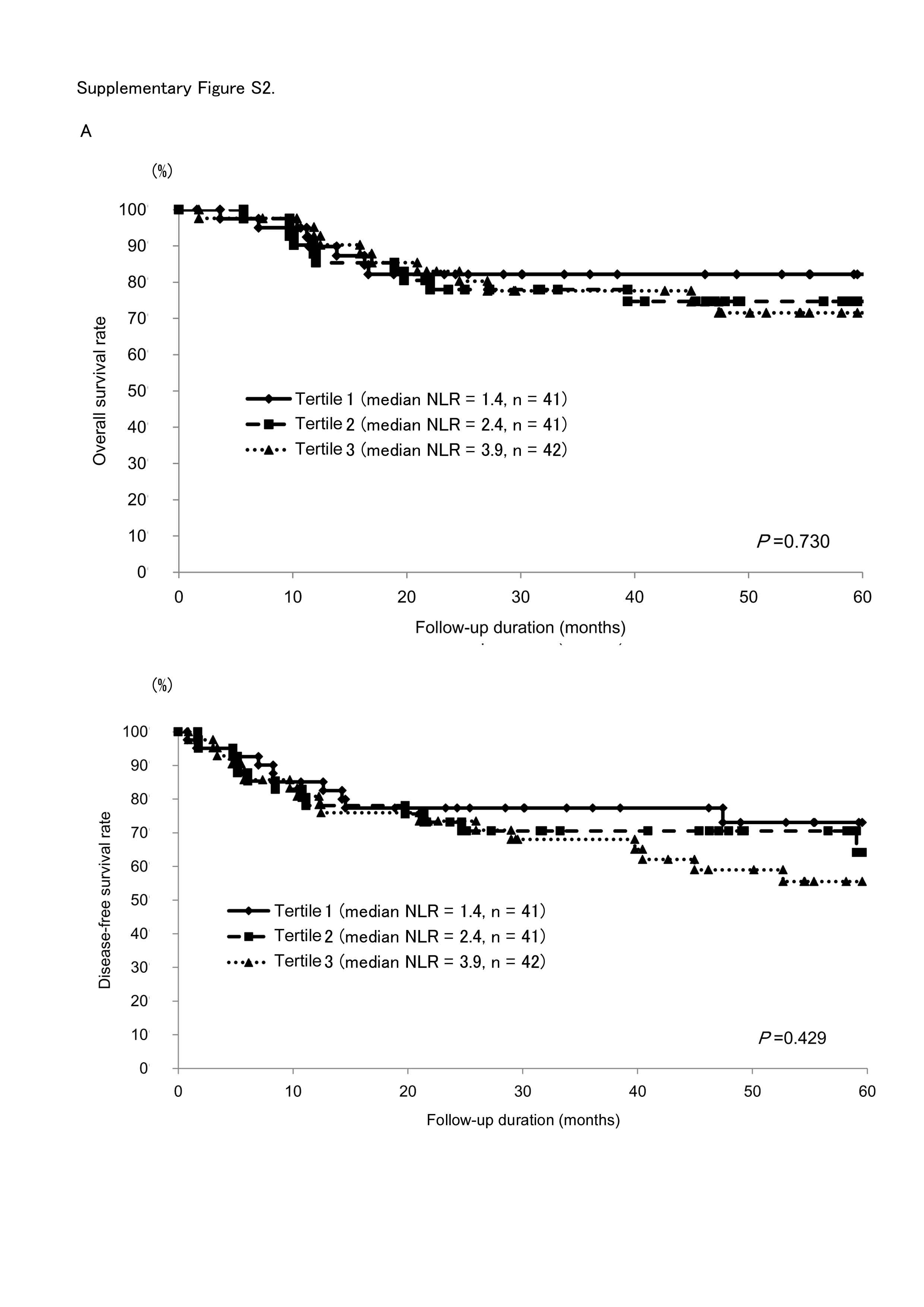

Supplement: Additional file 2: Figure S2. — The relationship between the NLR status and cancer-specific survival in patients with OSCC. In the Kaplan-Meier survival analysis of patients with oral squamous cell carcinoma (OSCC), the patients were divided into three groups based on their NLR status (Tertiles 1, 2 and 3). (A) The overall survival (OS) of the 124 OSCC patients stratified by their NLR status. (B) The disease-free survival (DFS) of the 124 OSCC patients stratified by their NLR status. (JPG 974 kb) [file 12885_2016_2079_MOESM2_ESM.jpg]
